# WALANT for distal radius fracture: open reduction with plating fixation via wide-awake local anesthesia with no tourniquet

**DOI:** 10.1186/s13018-018-0903-1

**Published:** 2018-08-06

**Authors:** Ying-Cheng Huang, Chien-Jen Hsu, Jenn-Huei Renn, Kai-Cheng Lin, Shan-Wei Yang, Yih-Wen Tarng, Wei-Ning Chang, Chun-Yu Chen

**Affiliations:** 1 Department of Orthopedics, Kaohsiung Veterans General Hospital, No. 386, Dazhong 1st Rd., Zuoying Dist., Kaohsiung City, 81362 Taiwan, Republic of China; 2 Department of Orthopedic Surgery, National Defense Medical Center, Taipei, Taiwan, Republic of China; 3 Department of Occupational Therapy, Shu-Zen Junior College of Medicine and Management, Kaohsiung, Taiwan, Republic of China

**Keywords:** Distal radius fracture, WALANT, Wide awake, No tourniquet

## Abstract

**Background:**

The wide-awake local anesthesia no tourniquet (WALANT) technique is applied during various hand surgeries. We investigated the perioperative variables and clinical outcomes of open reduction and internal fixation (ORIF) for distal radius fractures under WALANT.

**Methods:**

From January 2015 to January 2017, 60 patients with distal radius fractures were treated, and 24 patients (40% of all) were treated with either a volar or a dorsal plate via WALANT procedure. Of these 24 patients, 21 radius fractures were fixed with a volar plate, and the other 3 were fixed with a dorsal plate. Radiographs; range of motions; visual analog scale (VAS); quick disabilities of the arm, shoulder, and hand (Quick DASH) questionnaire; and time to union were evaluated.

**Results:**

One of the 24 patients could not tolerate the WALANT procedure and was reported as a failed attempt at WALANT. In the cohort, 23 patients successfully received distal radius ORIF under WALANT procedure. The average age is 60.9 (range, 20–88) years. The average operation time was 64.3 (range, 45–85) minutes, the average blood loss was 18.9 (range, 5–30) ml, and the average of duration of hospitalization is 1.8 (range, 1–6) days. The average postoperative day one VAS was 1.6 (range, 1–3). The average time of union was 20.7 (range, 15–32) weeks. The mean follow-up period was 15.1 (range, 12–24) months. Functional 1-year postoperative outcomes revealed an average Quick DASH score of 7.60 (range, 4.5–13.6) and an average wrist flexion and extension of 69.6° (range, 55–80°) and 57.4° (range, 45–70°). There was no wound infection, neurovascular injury, or other major complication noted.

**Conclusions:**

WALANT for distal radius fracture ORIF is a method to control blood loss by the effects of local anesthesia mixed with hemostatic agents. Without a tourniquet, the procedure prevents discomfort caused by tourniquet pain. Without sedation, patients could perform the active range of motion of the injured wrist to check if there is impingement of implants. It eliminates the need of numerous preoperative examinations, postoperative anesthesia recovery room care, and side effects of the sedation. However, patients who are not amenable to the awake procedure are contraindications.

**Electronic supplementary material:**

The online version of this article (10.1186/s13018-018-0903-1) contains supplementary material, which is available to authorized users.

## Background

Many minor procedures of the hand and wrist, such as carpal tunnel release and trigger finger release, could be performed with local anesthesia without sedation and could even be performed safely on an outpatient basis, but the common need of a tourniquet can cause pain and discomfort without general anesthesia or brachial plexus block. More recently, a newer technique called wide-awake local anesthesia no tourniquet (WALANT) in which lidocaine and epinephrine are injected for local anesthesia and vasoconstriction, respectively, has been increasingly used by hand surgeons [1, 2]. This technique enables the surgery to be performed with the patient fully awake and without a tourniquet, which allows intraoperative assessment of function during surgery.

Distal radius fracture is a common fracture associated with high-energy trauma in young adults or osteoporotic injury in the elderly [3, 4]. The use of a locking plate to treat such fractures has gained favor recently and helps maintain anatomical structure and facilitate earlier return to normal daily activities [5, 6]. However, plating for distal radius fractures usually requires more surgical time than that for minor hand surgeries as well as a bloodless surgical field to achieve the anatomical reduction. Typically, a tourniquet is used to minimize blood loss, and because of the long duration of surgery and tourniquet-related patient discomfort, open reduction and internal fixation (ORIF) has classically been performed under general anesthesia or brachial plexus block.

We have been using the WALANT technique since January 2015 to perform plating for distal radius fracture, but at present, there is no relevant literature on WALANT for this purpose. The purpose of this retrospective study was to investigate the perioperative variables and clinical outcomes of open reduction and internal fixation (ORIF) for distal radius fractures under WALANT technique, including the operation time, the blood loss, the duration of hospitalization, the time of union, the range of motion of the diseased wrist, the pain, and the functional outcome score 1 year postoperatively.

## Methods

From January 2015 to January 2017, 60 patients with distal radius fractures were treated. We excluded patients with concomitant injuries that needed further operative procedure under general anesthesia or spinal anesthesia, such as long bone fracture and traumatic cerebral hemorrhage, and those with peripheral vascular disease or allergy to lidocaine. However, cerebral hemorrhage not requiring surgical intervention was not considered a contraindication to WALANT, and these patients were still therefore included. The other reasons for not participating in the study group included the patient is willing for general anesthesia and the patients who felt lots of anxiety.

The patients were counseled clearly before consenting WALANT surgery. Before the decision of WALANT surgery, the patients were told honestly that the WALANT procedure required at least five needle punctures during the administration of anesthesia. The whole procedure and information about the composite of local injection solution including hemostatic agent were clearly explained.

Regarding other classical anesthesia options, the pros and cons were also informed. The use of sedation of general anesthesia might give rise to the postoperative vomiting and nausea (PONV), and the tourniquet pain and PONV during the recovery time might need extra consumption of medication for symptomatic control. Regional anesthesia like ultrasound-guided axillary brachial plexus block performed by the anesthesiologist for distal radius fracture ORIF is technically demanding, and the risk of unintentional intravascular injection or nerve injury was also told. The WALANT procedure was purely performed by an orthopedist, which saved the use of sedation and tourniquet, as well as lowered the risk of nerve injury.

**Fig. 1:**
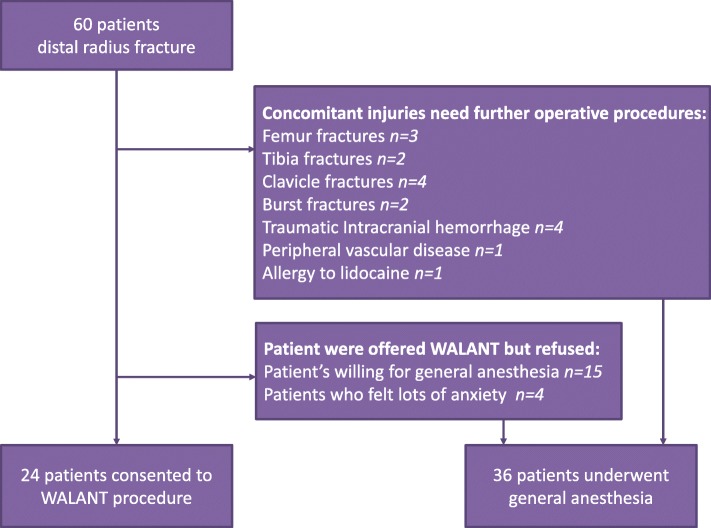
Flowchart of the inclusion and exclusion of participants in the current analysis

There were 17 patients with concomitant injuries needing further operative procedures. Nineteen patients were offered WALANT procedure but refused, and the reason for not participating in the study group included the patient is willing for general anesthesia (*n* = 15) and the patients who felt lots of anxiety (*n* = 4). Finally, 24 patients (40%) with distal radial fractures consented to WALANT surgery via fixation with volar plating or dorsal plating, and the other 36 patients were treated under general anesthesia (Fig. 1). The institutional review board of Kaohsiung General Veterans Hospital approved this retrospective study (IRB number: VGHKS17-CT8-13), and informed consent was obtained from all patients.

Anteroposterior and lateral wrist radiographs were obtained on the first admission following trauma. All fracture patterns were recorded according to the Arbeitsgemeinschaft für Osteosynthesefragen/Orthopedic Trauma Association (AO/OTA) fracture classification system.

The indication for using volar plate fixation and dorsal plate depended on the fracture pattern and the surgeon’s preference. According to the AO/OTA fracture classification system, of all the patients, 10 cases were classified as extra-articular fracture (6 cases of A2 type, 4 cases of A3 type), and the other 14 cases were intra-articular fractures (3 cases of B2 type, 5 cases of B3 type, 2 cases of C1 type, 3 cases C2 type, and 1 case of C3 type) (Table 1).

**Table 1:** Demographic data of the patients and their radiological and clinical outcomes

No.	Age	Sex	Side	AO/OTA classification	Type of approach	OP time (min)	Blood loss (ml)	Duration of hospitalization (days)	Time to union (weeks)	Flexion (degrees)	Extension (degrees)	Postop d1 VAS	Quick DASH score	Follow-up period (months)	Remarks
1	69	F	L	C2	Volar plating	75	5	2	16	60	50	3	4.5	14	
2	57	F	R	A2	Volar plating	60	10	5	16	75	65	1	4.5	20	Subdural hemorrhage
3	71	F	R	B3	Volar plating	45	5	1	24	80	65	2	6.8	12	
4	64	F	L	B2	Dorsal plating	65	20	1	22	75	65	1	9.1	17	
5	20	M	L	B3	Volar plating	75	10	2	20	70	60	1	4.5	18	
6	70	F	R	C2	Volar plating	60	20	1	16	60	45	3	4.5	13	
7	88	F	R	A2	Volar plating	55	10	1	24	75	60	1	6.8	14	
8	63	F	R	A2	Volar plating	90	50	2	24	70	65	3	11.4	13	Shift to general anesthesia
9	80	M	L	A2	Volar plating	70	20	2	28	75	60	1	9.1	24	
10	40	M	R	B3	Volar plating	70	30	1	32	55	55	1	6.8	12	
11	46	F	R	B3	Volar plating	60	20	1	20	75	70	1	4.5	14	
12	65	M	L	C1	Volar plating	60	30	2	16	70	50	3	6.8	16	Distal ulna fracture
13	70	F	R	B2	Dorsal plating	85	20	1	22	70	60	1	6.8	15	
14	76	F	R	B2	Dorsal plating	65	25	2	20	75	45	1	9.1	12	
15	73	F	L	A3	Volar plating	70	30	1	16	70	60	3	9.1	12	
16	46	M	L	B3	Volar plating	60	20	6	18	60	65	1	11.4	24	Subdural hemorrhage
17	65	F	L	C1	Volar plating	60	30	2	24	65	45	2	11.4	12	
18	70	M	R	A3	Volar plating	85	20	1	22	70	50	2	9.1	15	
19	76	F	R	C2	Volar plating	65	25	2	15	60	55	1	9.1	15	
20	73	F	L	A3	Volar plating	70	30	1	20	80	60	2	4.5	15	
21	23	M	R	A2	Volar plating	55	10	3	24	80	70	1	4.5	18	Multiple rib fractures
22	26	M	R	A2	Volar plating	50	5	2	22	65	50	2	6.8	12	
23	57	M	L	C3	Volar plating	70	10	1	20	60	45	2	13.6	12	
24	75	F	R	A3	Volar plating	50	30	1	20	75	70	1	11.4	12	

*VAS* visual analog scale, *Quick DASH* quick disabilities of the arm, shoulder, and hand, *ORIF* open reduction internal fixation, *CRIF* close reduction internal fixation

In our study, 24 patients were treated with either one or the other. Twenty-one patients were treated with volar plate fixation. Only three patients were treated with dorsal plate fixation, whose distal radius fractures contained the small fragment of the dorsal rim and needed definite dorsal buttress so as to avoid radiocarpal subluxation. Regarding the volar column plate implants, 18 cases used the Synthes 2.4-mm LCP Distal Radius System (DePuy Synthes; Johnson & Johnson Family of Companies, MA, USA), 2 cases used the Civic Locking Plate and Screw System (Microwave Precision, Taiwan), and 1 case used the Aplus Distal Radius Locking Plate System (APlus Biotechnology Corporation, Taiwan). On the other hand, dorsal plating using the 2.4-mm LCP Distal Radius System with a right angle L-shaped plate (DePuy Synthes; Johnson & Johnson Family of Companies, MA, USA) was performed in three cases.

### Surgical procedure: preparation

**Fig. 2:**
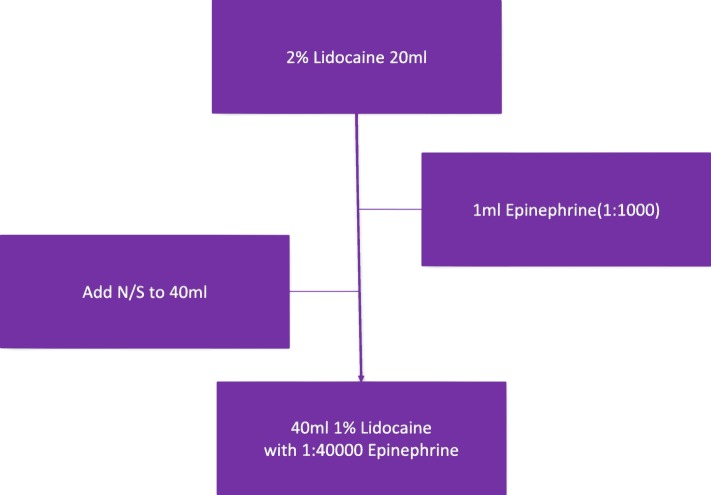
The solution used in WALANT technique

In our institution, the solution used in the WALANT technique consisted of 1 ml of epinephrine (1:1000) and 20 ml of 2% lidocaine, which were mixed with normal saline to give a total of 40 ml, that is, the solution of 1% lidocaine mixed with 1:40000 epinephrine for later injection (Fig. 2). A set of baseline parameters, including heart rate, blood pressure, respiratory rate, and oxygen saturation, was obtained during the entire surgery. At the same time, preoperative intravenous antibiotics with 1 g cefazolin were given for each patient as prophylaxis. The amount of blood loss was based upon the amount in a suction container in the operation room.

### Surgical procedure: hematoma block and local anesthetic injection

**Fig. 3:**
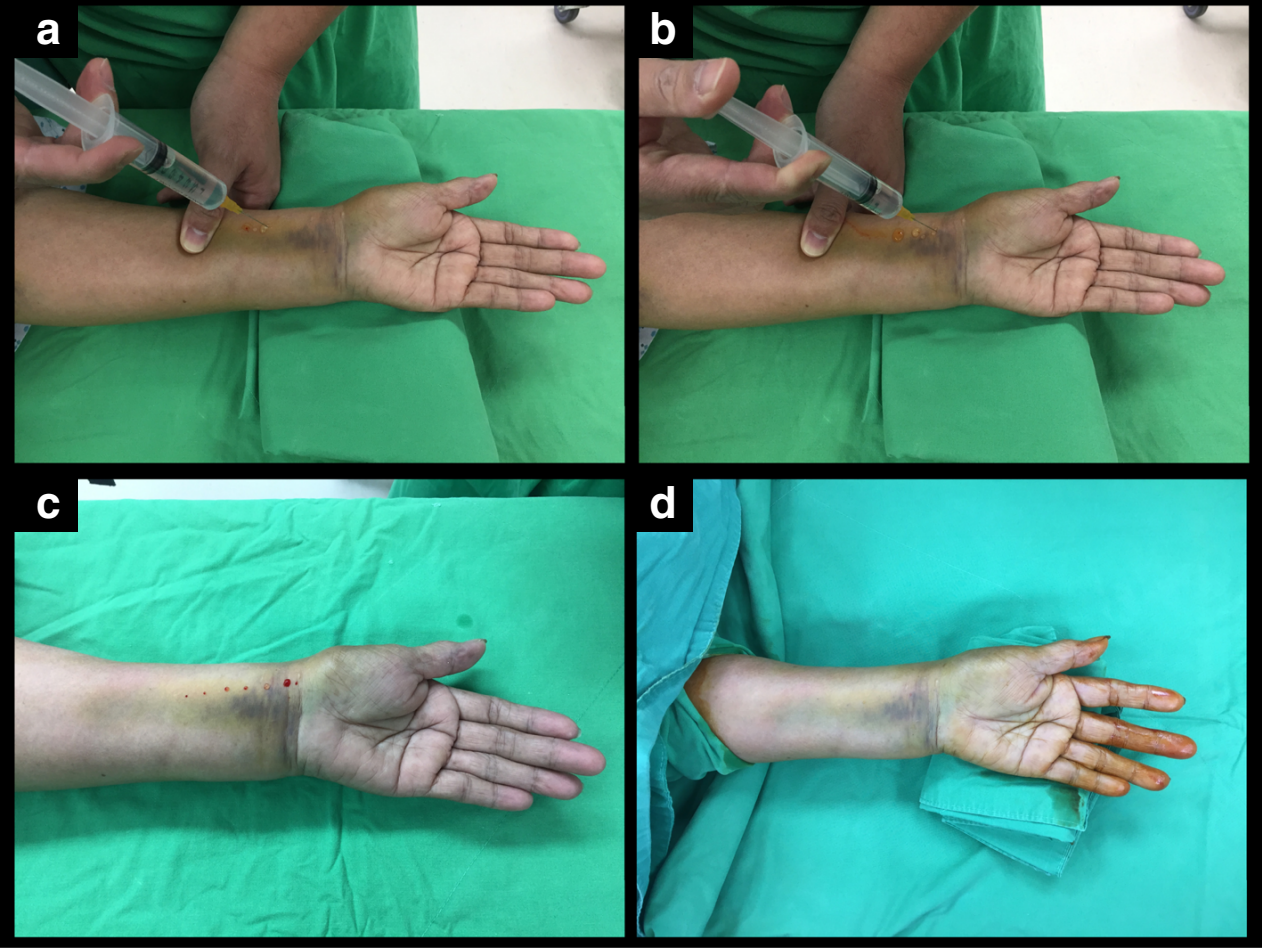
Subcutaneous injection for volar plating. **a** 1% lidocaine mixed with 1:40000 epinephrine for local anesthesia. **b** Injection from proximal to distal wherever any incision. **c** After injection, the injured forearm was sterilized and prepared for operation and wait for the hemostatic effect. **d** The optimal hemostatic effect was achieved about 18 min after

The protocol using the WALANT technique in our case series started with a hematoma block via a 3–5 ml 1% lidocaine injection from the dorsal site into the fracture site to minimize the discomfort due to sterilization and manipulation procedures for the fractured wrist [7–10]. Subcutaneous injection with 1% lidocaine mixed with 1:40000 epinephrine approximately 5–10 ml was administered directly onto the operative volar or dorsal region of the distal radius. A needle smaller than 25 G was used to perform subcutaneous injection to minimize the injection discomfort (Fig. 3). Then, the injured forearm was sterilized and prepared for surgery while the surgeons waited approximately 18 min to perform the incision for a good hemostatic effect.

#### Volar plating

**Fig. 4:**
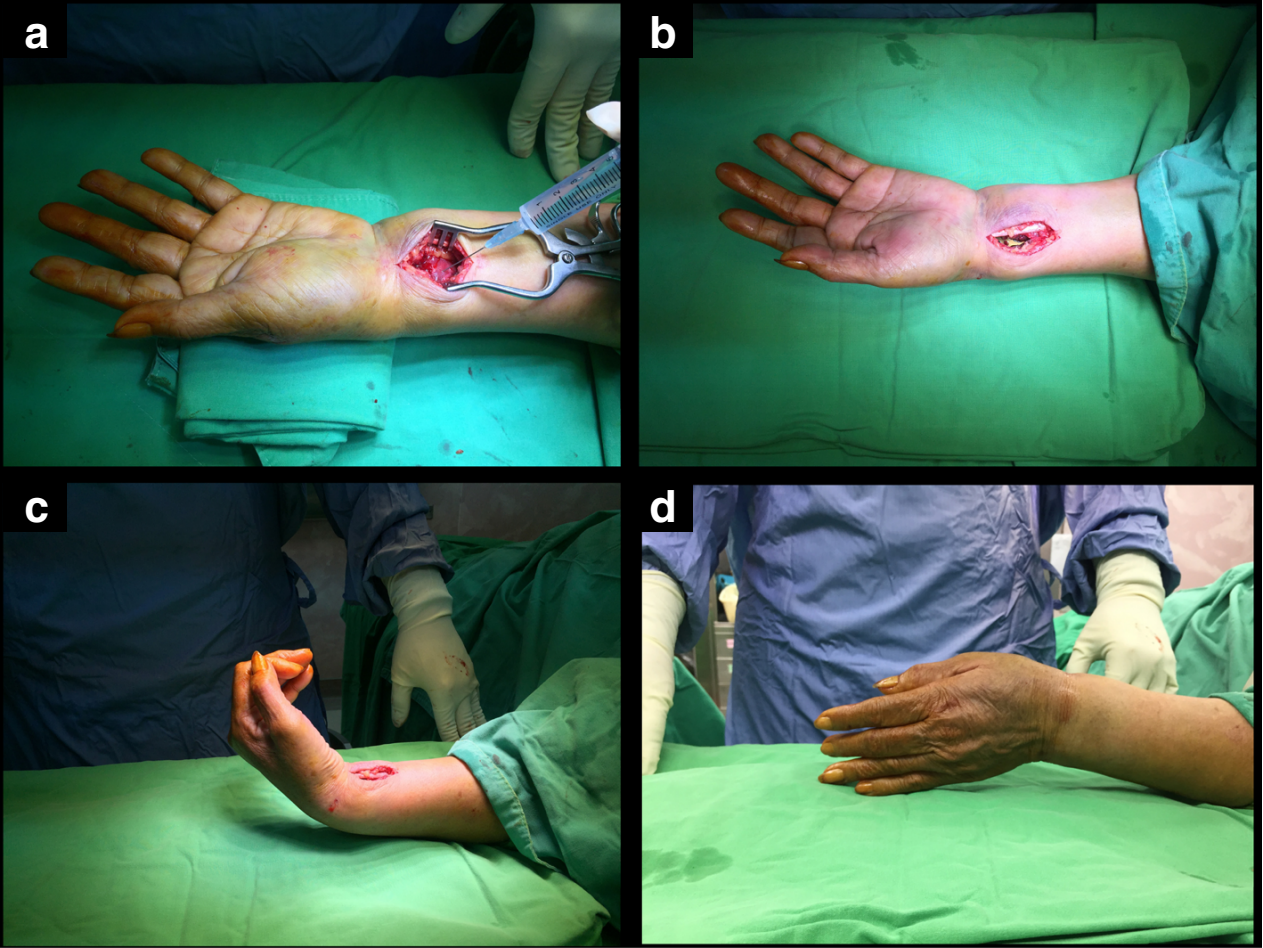
Henry approach via WALANT technique. **a** Before splitting the pronator quadrates, we injected an additional 5 ml of 1% lidocaine mixed with 1:40000 epinephrine beneath it for later procedures. **b** Volar ORIF with plating. **c** The patient was required to perform active range of motion with wrist extension and flexion. **d** Perform radial and ulna deviation

The procedure started with a longitudinal skin incision approximately 4 to 5 cm along the flexor carpi radialis (FCR) tendon. The sheath was opened, and the FCR was retracted toward the ulna to deepen the incision between the flexor pollicis longus and radial artery to achieve exposure of the pronator quadratus (PQ) [11]. An additional 5 ml of 1% lidocaine mixed with 1:40000 epinephrine was injected beneath the PQ, and the surgery halted about 30 s while the local anesthetic took effect within the PQ, which was later split and elevated for fracture reduction, plate placement, drilling procedures, and screw fixations (Fig. 4).

#### Dorsal plating

Using a single dorsal skin incision along the line between the third and fourth compartments, the extensor retinaculum was carefully dissected. An additional 5 ml of 1% lidocaine mixed with 1:40000 epinephrine was injected beneath the retinaculum to infiltrate the tendon sheath and the periosteum of the intermediate column. Then, the extensor retinaculum was opened as far as needed for later fracture reduction, plate placement, drilling procedures, and screw fixations.

### Postoperative care and follow-up

Postoperative treatment following the surgical procedure for the patients was standardized. Regular oral tramadol 37.5 mg/acetaminophen 325 mg combination tablets (Ultracet®) two times a day was the protocol for postoperative pain control medication for each patient (usually about 10 days after operation). Immobilization with a short arm splint for 1 week was performed for each patient. The patients were taught to use a consecutive passive wrist motion with flexion and extension from the removal of the splint to postoperative 1 month, under weekly outpatient department close follow-up. Then, active training of a range of motion (ROM) using objects weighing up to 5 kg was started since the second month postoperatively. There was no implant removal after surgery during follow-up of these patients. All patients performed exercise on their own and were followed up for ≥ 12 months postoperatively.

According to the clinician, the patients achieved clinical union without pain or any clinical symptom postoperatively. Anteroposterior (AP) and lateral wrist radiographs were obtained for the radiographic evaluation of the bone union, in which the fracture gap disappeared on the AP and lateral views, which was also confirmed by the radiologist’s report of the plain film at the same time. The functional outcomes by using the quick disabilities of the arm, shoulder, and hand (Quick DASH) questionnaire [12] were evaluated 1 year postoperatively, and the maximum ROM at the wrist was also recorded at our outpatient department monthly.

## Results

The demographic information, perioperative variables, and clinical outcomes are presented in Table 1. One of the 24 patients could not tolerate the WALANT procedure and was reported as a failed attempt at WALANT. Regarding the other 23 patients under WALANT procedure, the average age is 60.9 (range, 20–88) years. Concerning the time from injury to surgery, 7 patients were arranged for the operation on the same day, and the other 16 patients received the procedure on the next day. The average surgical time was 64.3 min (range, 45–85 min). The average amount of blood loss during surgery was 18.9 ml (range, 5–30 ml). The average duration of hospitalization was 1.8 days (range, 1–6 days). The average time to union for each radius was 20.7 weeks (range, 15–32 weeks). The average follow-up period was 15.1 months (range, 12–24 months).

The patient who could not endure the subcutaneous injection and felt very nervous under an awake status agreed to receive general anesthesia to complete the surgery, which was still performed without a tourniquet. The operation time of the patient was more than the average of the other 23 patients under WALANT procedure (90 vs. 64.3 min).

In our study, there was a victim of multiple rib fractures, two minimal subdural hemorrhages not requiring surgical intervention, one patient with a distal radius and ulna fracture receiving percutaneous pinning for distal ulnar fracture in addition to volar plating of the radius. Despite the associated injuries, they still underwent the WALANT procedure for distal radius fracture fixation successfully.

The average postoperative day 1 visual analog scale score was 1.6 (range, 1–3). Postoperative pain control medication is mainly Ultracet 500 mg two times a day during hospitalization, as well as the medication sent home with. During outpatient department follow-up, almost all the patients undergoing WALANT procedure claimed that no extra medication for pain was needed.

The average Quick DASH score of the 23 injured radii was 7.60 (range, 4.5–13.6). The patient with the highest Quick DASH score of 13.6 was a 57-year-old male with a C3-type distal radius fracture. It was particularly difficult for him to turn a key and use a fork with his injured wrist. The average flexion and extension of each wrist were 69.6° (range, 55–80°) and 57.4° (range, 45–70°), respectively.

During the follow-up period, none of the patients received secondary interventions, such as bone grafting or shockwave treatment, and all patients achieved union. No wound infection, finger necrosis, palpitation, or other complication was found in our study.

## Discussion

The use of a tourniquet for hemostatic effect has been popular in simple hand surgeries, such as tendon repair or transfer, transverse carpal ligament release, and full-thickness skin grafts. However, the patients could not tolerate very long operation time because of tourniquet pain. Hutchinson et al. and Maury et al. reported studies about the tourniquet tolerance among healthy volunteers, in which the average tolerance to forearm tourniquet was 13 and 25 min, respectively, with temporary pain and paresthesia [13, 14]. Prolonged tourniquet usage may give rise to nerve injury or severe neurological deficit [15]. Thus, a wide-awake local anesthesia with no tourniquet procedure was developed. The WALANT technique has been used in different hand surgeries, particularly in soft tissue repair or reconstruction [1, 16–20]. In addition, finger fractures could be also treated by closed reduction and percutaneous pinning under WALANT technique [21]. The WALANT technique has several advantages, including simplifying the preparations for surgeries, lowering the risk of general anesthesia, and saving time in the postoperative recovery room. Furthermore, it may reduce medical costs of preoperative evaluation of general anesthesia, shorten hospitalization days, decrease opioid agent consumption, and save medical resources [16]. To our knowledge, no study has been reported regarding the management of distal radius fractures by using ORIF with the WALANT technique.

In previous studies, Lalonde reported that the effective concentration of the hemostatic agent is from 1:400000 epinephrine in treating tendon repair on wrist surgery to 1:100000 epinephrine in surgical dissection and manipulation for fractured bones [16]. The optimal time delay between local injection and incision to minimize bleeding depends on the maximal vasoconstriction [22], occurring approximately 25.9 min after injection of 1:100000 epinephrine beneath the skin [23]. In the present study, we performed the surgeries over the wrist not only for soft tissue dissection but also for bony procedures, such as reduction with forceps and manipulation with limb traction. Unlike treating finger fractures which may only need fixation with percutaneous pinning or open reduction at a small area, orthopedic surgeons had to manage blood loss due to the transection through the subcutaneous vessel from a relatively larger operation field as well as bleeding from the bone marrow in the fracture sites. Therefore, the regimen of the WALANT technique in our study for distal radius fractures is 1:40000 epinephrine, which is more concentrated than that reported in previous studies to meet the hemostatic effects during the whole operation [16]. The time delay between the local injection and incision in our study was 18 min, which provided sufficient time for the medical staff to sterilize the diseased limb before the surgeons’ incision and achieve a hemostatic effect during the whole operation. Otherwise, after the initial hematoma block combined with preventive injection underneath the PQ muscle, bony procedures, such as drilling and screwing into bones, could be performed without any discomfort.

The safety of epinephrine use in hand surgeries has been previously established [24]. Cyanosis of the distal fingers due to epinephrine use has been previously reported, but procaine acidity was found to be the culprit for finger loss [25, 26]. Even when procaine has been confirmed as the real cause of necrosis, there still have been concerns about the possible vasoconstriction caused by epinephrine in the human finger, and phentolamine has been used to reverse the vasoconstriction in rare cases of necrosis onset. Nodwell and Lalonde reported that the white finger can be reversed by subcutaneous injection of 1 mg of phentolamine in 220 cc of saline wherever the epinephrine is injected [27]. Certainly, this situation seldom occurs. However, if there is a suspicion of cyanosis of the distal finger related to the epinephrine use, the vasoconstriction can be reversed. In our study, we injected the more concentrated epinephrine cocktail into the distal radius area instead of the distal fingers, and there was no remarkable ischemia sign or finger necrosis. Epinephrine-induced cardiac ischemia has been rarely reported, even with a high dose of 1:1000 epinephrine [28]. In our study, no complication such as finger necrosis, palpitation, or allergy was found.

Without tourniquet use, hemostatic control could still be achieved, and no patient received a blood transfusion in our study. The average blood loss was only 18.9 ml. In fact, such a small amount of blood loss does not interfere with the whole procedure. In Ruxasagulwong’s prospective trial [29] regarding common minor orthopedic hand soft tissue surgery (including carpal tunnel syndrome, de Quervain’s disease, and trigger finger), even though there was a more surgical field bleeding in the wide-awake group without tourniquet application, the amount of blood loss in the conventional group with tourniquet use was significantly higher because of vasodilatation with a moderate amount of bleeding after the release of tourniquet pressure prior to skin suture to check for bleeding. It is worth mentioning that the amount of blood loss under the WALANT technique may be relatively less than the uncalculated blood loss after tourniquet release.

Lalonde et al. reported that carpal tunnel release and trigger finger release were minor surgeries, which could be performed in procedure rooms under field sterility with very low infection rates [2, 30]. In our hospital, the fracture fixation under WALANT technique was still performed in the main operation room with well-equipped tools and image intensifier. Preoperative intravenous antibiotics with 1 g cefazolin were still given to each patient as prophylaxis for the purpose of infection prevention. In addition, all patients stayed in the hospital at least one night, and we could teach the patients how to care for the surgical wound. Therefore, no wound infection was encountered in our study.

When we performed minor orthopedic hand soft tissue surgery such as trigger finger release, the patient is asked to performed metacarpal-phalangeal joint flexion to confirm the adequate release of A1 pulley. Similarly, when distal radius ORIF under WALANT technique was performed, the patient was asked to performed wrist and finger motions to confirm the stability of fracture fixation and to see if there was an impingement of implant (Additional files 1 and 2: Videos S1 and S2).


**Additional file 1: Video S1.** Active motion after volar plating via WALANT procedure. There was no impingement of implant while the patient performed wrist motion. (MP4 14211 kb)



**Additional file 2: Video S2.** Active motion after dorsal plating via WALANT procedure. The patient performed all finger extension smoothly, including extensor pollicis longus tendon. (MP4 10004 kb)


Health costs have continued to increase over time [31]. Assessments of cost savings and patient satisfaction also have been reported and need to be discussed [32]. Furthermore, the National Health Insurance Administration-Ministry of Health and Welfare has already started bundling payments of selected orthopedic diagnosis-related groups (DRG) in Taiwan for a period of time. The hospitals receive a single index code with the same payment for a particular DRG, in order to reduce the cost as possible. One of the main costs for the payment is the stays of patient’s hospitalization. In the present study, the average operation time was 64.3 min (range, 45–85 min), and the average of duration of hospitalization was 1.8 days (range, 1–6 days). Two patients with subdural hemorrhage had relatively longer hospital stays than the average (5 and 6 days, respectively), because they needed more days for the complete observation of head injuries. For cost efficiency, WALANT eliminates the need for sedation, which means that there is no need for numerous preoperative examinations, specialists monitoring intraoperative sedation, postoperative anesthesia recovery room care, and that there will be less incidence of nausea, vomiting, or unwanted side effects of opiates or sedation. Dr. Lalonde claimed that patients could spend less time at the hospital for the procedure because there is no anesthesia recovery time, and the patients could talk to their surgeon during the WALANT procedure for advice on how to avoid complications, when to return to work, and how to take pain medication. Additionally, the patients do not need preoperative anesthetic assessment visits, chest X-rays, and needle pricking for blood tests, and the patients do not need to fast prior to general anesthesia, change medication schedules (such as required for patients with diabetes under regular oral hyperglycemic drug control), discontinue anticoagulation medication, or a caregiver during the evening shift [33].

Some patients, such as uremia patients with ipsilateral wrist fractures and arteriovenous shunts who should not be subjected to tourniquet applications, those with chronic occlusive pulmonary disease or severe congestive heart failure and other cardiovascular problems with difficulties in extubation after general anesthesia, may benefit from surgeries under the WALANT technique. In addition to the benefits mentioned above, doctors can perform surgery with the patient awake and directly examine them for active ROM and tendon function of the injured limbs and reduce the rate of impingement or of usual complications, such as tendon irritation (even that caused by compression of the plate), which cannot be achieved under general anesthesia.

Discussions with the patients were made regarding the rehabilitation plans, to provide education concerning wound care, and about the return to work after surgery, which increases patient’s sense of safety and builds their confidence for achieving a good recovery [34]. Adequate explanations about the whole procedure are necessary for patients who are about to receive distal radius ORIF under the WALANT technique. In our case series, even after we fully discussed the discomfort caused by local injection before surgery, one patient still felt very unwell and requested shifting to general anesthesia. Therefore, surgeons should evaluate the patient’s personality prior to the WALANT technique for ORIF of distal radius because patients with psychological problems or lots of anxiety are contraindications for the WALANT procedure. For those who are not amenable to the awake procedure, ORIF of distal radius under general anesthesia is still recommended.

This study had several limitations, including its retrospective nature and small sample size. In addition, there was no control group of patients treated with general anesthesia without a tourniquet. Further comparative analyses of distal radius ORIF under the WALANT technique and under general anesthesia without a tourniquet with a large group of consecutive patients are warranted.

## Conclusions

Patients receiving the WALANT surgery for distal radius ORIF do not require sedation, which allows the patients to communicate with the doctors during the procedure and perform active movement of the operated limb to examine if there is an impingement of implants, as well as save the need of numerous preoperative examinations, specialists monitoring intraoperative sedation, postoperative anesthesia recovery room care, and the side effects of opiates or sedation. Without tourniquet use, tourniquet pain can be avoided and blood loss can be controlled by epinephrine injection. No complications such as infection or implant failure were observed in our case series. Most importantly, a sufficient explanation of the whole procedure is necessary for patients who are about to receive distal radius ORIF under the WALANT technique. Patients who are not amenable to the awake procedure are contraindications.

## Abbreviations

DRG: Diagnosis-related groups
GA: General anesthesia
WALANT: Wide-awake local anesthesia no tourniquet
